# “Add More Arrows to Your Quiver”: The Role of Adding Another Chemotherapy Drug to Fluoropyrimidine and Long Term Radiotherapy in Locally Advanced Rectal Cancer: A Systematic Review and Meta-Analysis

**DOI:** 10.3390/jcm14020345

**Published:** 2025-01-08

**Authors:** Jacopo Giuliani, Umberto Tebano, Marta Mandarà, Antonella Franceschetto, Carlotta Giorgi, Sonia Missiroli, Milena Gabbani, Giuseppe Napoli, Nicoletta Luca, Daniela Mangiola, Marco Muraro, Mariasole Perrone, Paolo Pinton, Francesco Fiorica

**Affiliations:** 1 Department of Clinical Oncology, Section of Medical Oncology, AULSS 9 Scaligera, 37045 Legnago, Italy; jacopo.giuliani@aulss9.veneto.it (J.G.); marta.mandara@aulss9.veneto.it (M.M.); daniela.mangiola@aulss9.veneto.it (D.M.); 2 Department of Clinical Oncology, Section of Radiation Oncology and Nuclear Medicine, AULSS 9 Scaligera, 37045 Legnago, Italy; umberto.tebano@aulss9.veneto.it (U.T.); antonella.franceschetto@aulss9.veneto.it (A.F.); milena.gabbani@aulss9.veneto.it (M.G.); giuseppe.napoli@aulss9.veneto.it (G.N.); nicoletta.luca@aulss9.veneto.it (N.L.); marco.muraro@aulss9.veneto.it (M.M.); 3 Department of Morphology, Surgery and Experimental Medicine, Section of Pathology, Oncology and Experimental Biology, Laboratory for Technologies of Advanced Therapies (LTTA), University of Ferrara, 48033 Ferrara, Italy; grgclt@unife.it (C.G.); msssno@unife.it (S.M.); prrmsl@unife.it (M.P.); paolo.pinton@unife.it (P.P.)

**Keywords:** intensified chemotherapy, chemoradiotherapy, neoadjuvant therapy, rectal cancer, radiotherapy, chemotherapy

## Abstract

**Objectives:** Despite optimal local control obtained with neoadjuvant chemoradiotherapy (CRT), data on overall survival (OS) and disease-free survival (DFS) of local advanced rectal cancer patients are still equivocal. This meta-analysis aimed to estimate the pathological complete response (pCR), regression rate, DFS, and OS probabilities of rectal cancer patients treated with a second chemotherapy drug added to fluoropyrimidine and long-term radiotherapy. **Methods:** Computerized bibliographic searches of MEDLINE, PUBMED, Web of Science and the Cochrane Central Register of Controlled Trials databases (1970–2023) were supplemented with hand searches of reference lists. Studies were included if they were randomised controlled trials (RCTs) comparing intensified chemotherapy with CRT to preoperative CRT and if they had patients with resectable, histologically proven rectal adenocarcinoma without metastases. **Results:** Eighteen RCTs (7695 patients) were analysed. Data on population, intervention, and outcomes were extracted from each RCT, following the intention-to-treat method, by three independent observers and combined using the DerSimonian and Laird methods. A chemotherapy with two drug and long-term radiotherapy CRT, compared to preoperative CRT (fluoropyrimidine and long-term radiotherapy), significantly increases the rate of pathological complete response (OR 1.37 (95% CI, 1.16–1.63) *p* = 0.0003) and the regression rate (OR 1.57 (95% CI, 1.16–2.14) *p* < 0.00001). Furthermore, it increases DFS (HR 0.87 (95% CI, 0.79 to 0.95) *p* = 0.002 and OS HR 0.84 (95% CI, 0.74 to 0.95) *p =* 0.007). The risk of severe adverse events (≥G3) is increased OR 1.96 (95% CI 1.35–2.85), *p* = 0.0005. **Conclusions:** In patients with resectable rectal cancer, intensified chemotherapy can reduce by 13% the risk of disease progression and by 16% the risk of death.

## 1. Introduction

Worldwide, colorectal cancer (CRC) is widespread. Globocam estimates that the burden of CRC will increase to 3.2 million new cases and 1.6 million deaths by 2040 [1]. Treatment of locally advanced disease, particularly those of the rectum, is a matter of debate. In 2000, a meta-analysis demonstrated that preoperative radiotherapy significantly improved overall survival compared to surgery alone [2,3]. Surgical techniques have also evolved to improve outcomes for these patients, although only recently have these improvements been standardized. The introduction of the total mesorectal excision (TME) [4] technique has significantly improved local recurrence-free survival.

However, preoperative radiotherapy has demonstrated significant therapeutic gain [5,6] in all patients treated surgically with TME, including the elderly [7].

The gold-standard treatment, especially for low rectal tumours, is a neoadjuvant approach with long term radiotherapy, fluoropyrimidine, and TME surgery [8]. Many attempts have been made to improve its effectiveness ulteriorly. If, with this gold standard, only 5% of patients will have a local recurrence disease, there is no demonstrated benefit in decreasing the rate of distant metastasis, with data at five years of 32% [9].

It is necessary to reduce the rate of distant relapse. According to a cancer-centric vision, it is important to destroy as many tumour cells as possible, especially those that have already metastasized. In recent years, oncologists have increased the dosage of chemotherapy and/or radiation therapy to increase their effectiveness in destroying these cells. The results are always contrasting in all areas of application. There is no deterministic relationship between increasing cell lethality and improving outcomes. It is not surprising to find two rectal cancer patients with the same morphology and profile but with two completely opposite therapeutic results.

Evidently, there is something that is not working, and it is necessary to overcome this situation by considering how radiation and chemotherapy drugs interact not only with the tumour but above all with the microenvironment in which the tumour cells grow.

To change the natural history of rectal cancer and increase survival, it is necessary to take advantage of all the treatments used in randomized clinical trials and reinterpret these results with a new vision.

In this setting, the clinical question is if an interaction of long-term radiotherapy with combined chemotherapy drugs can reduce the distant recurrence rate and improve disease-free survival (DFS) and, hopefully, overall survival (OS).

We performed a meta-analysis of randomised clinical trial studies (RCTs) to increase statistical power, including patients with LARC who received different preoperative combined and intensified strategies. Our study achieved the following:

1. We estimated the pooled actuarial probabilities of disease-free survival, overall survival, local recurrence and distant metastases in LARC patients treated in these RCTs;
2. We analysed variabilities in OS by considering the heterogeneity between studies;
3. We identified factors associated with the risk of recurrence and survival.

## 2. Materials and Methods

### 2.1. Selection of Trials

This meta-analysis followed the PRISMA statement [10]. A systematic search of MEDLINE and the Cochrane Central Register of Controlled Trials databases was performed for articles published up to 31 December, 2022, with no lower date limit, including the following key “locally advanced rectal cancer”, “neoadjuvant chemoradiotherapy”, “total neoadjuvant treatment”, “induced chemotherapy”, “neoadjuvant radiotherapy”, and “randomised trial and clinical trial”. The reference lists of all retrieved review articles and primary studies were manually searched to identify additional studies. Conference proceedings (ASCO, ASTRO, ESMO, and ESTRO) were manually searched to ensure that the latest oncological data were considered. When the results of a single study were reported in more than one publication, the extrapolated results were used with priority given to those with the longest follow-up. Studies were included in the analysis if they were Phase III RCTs comparing neoadjuvant radiochemotherapy with intensified chemotherapy and long-term radiotherapy to the standard approach and if they had local advanced rectal cancer without extra pelvic disease.

**Figure 1:**
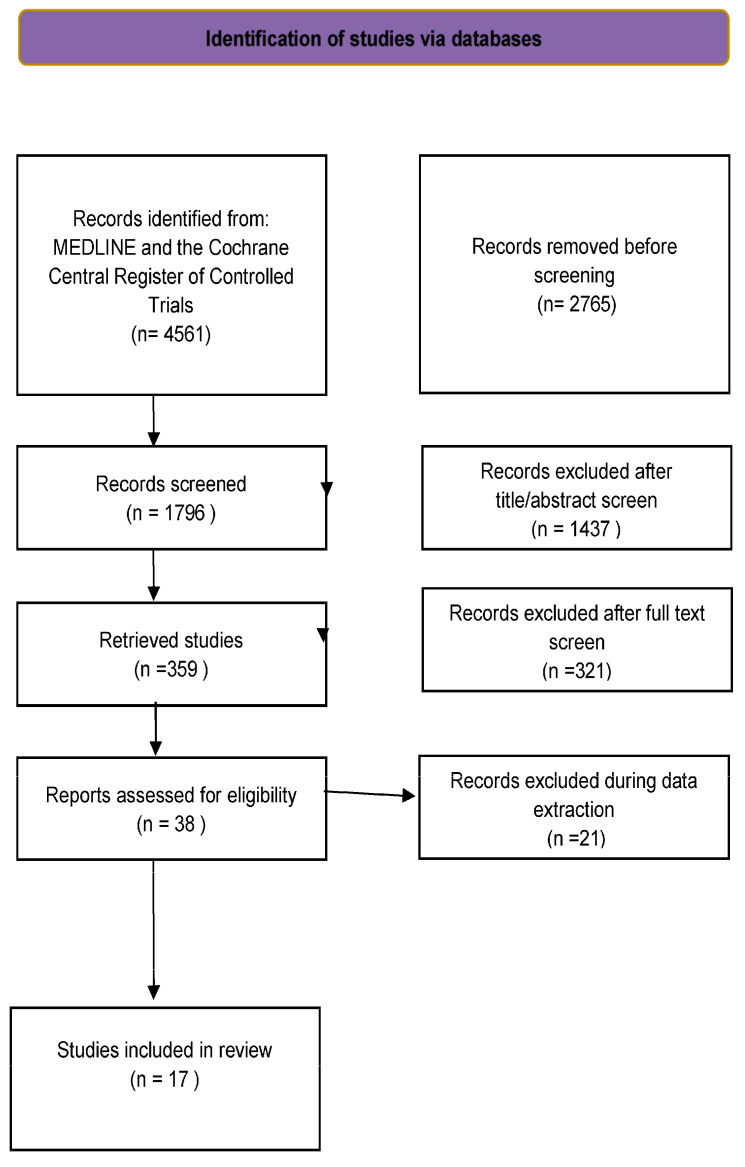
Study flow chart.

Among the 4803 studies reviewed, 17 (Figure 1) met the inclusion criteria.

### 2.2. Review of Studies

The trials were first reviewed using a list of predefined pertinent issues concerning patients’ characteristics and treatments. Study- and patient-level variables were extracted from all studies and entered into a database. Study-level variables included the study name, the first author’s last name, publication year, the region where the study was performed, the number of subjects, the number of centres (single versus multiple), outcomes measured, and study validity. Patient-level variables included mean age, sex, clinical stage, and oncological performance scale. Three independent investigators (CG, MM, and UT) evaluated and classified each RCT. Clinical outcomes were pathological response as complete response (pCR) rate and regression grade (from complete regression to fibrosis and tumour cells with a preponderance of fibrosis), disease-free survival (DFS), overall survival (OS), local recurrence-free survival, and distant metastasis-free survival. Discrepancies between reviewers were infrequent (overall interobserver variations < 10%) and were resolved by discussion. Two independent reviewers (GN, JG) assessed the risk of bias using the Cochrane Risk of Bias table [10]. This tool encompasses six domains: (1) random sequence generation, (2) allocation concealment, (3) blinding of participants/personnel, (4) blinding of outcomes assessors, (5) incomplete outcome data, (6) selective reporting of outcomes, and (7) other potential sources of bias. The study-level assessment was applied for domains 1, 2, 6, and 7, and the outcome-level assessment was applied for domains 3, 4, and 5 of each trial. A third investigator was consulted in case of disagreements (Figure S1).

### 2.3. Statistical Analyses

Pooled hazard ratios (HRs) for time-to-event outcomes (OS, DFS, local control, and distant metastasis) in the experimental and control arms were either extracted directly from the publications or estimated. The HR estimates were combined into meta-analyses by the inverse variance method. For dichotomous outcomes, crude rates of PCR, downstaging, and safety outcomes, differences observed between the two groups were expressed as the pooled odds ratio (OR), with its 95% confidence interval (CI). Both analyses assessed heterogeneity between studies using the Pearson χ^2^ test and the I^2^ statistic [11]. However, all treatment effects on the defined outcome measures were calculated using models based on random effect assumption. Begg’s funnel plots were generated, and Egger’s regression asymmetry test was used to examine potential publication bias related to DFS and OS. All these analyses were computed using (R Foundation for Statistical Computing, Vienna, Austria).

## 3. Results

More than 4500 records were retrieved from the computerized database search. After removing duplicates, 640 were considered for the title and abstract review. Eighty-two records were selected as potentially relevant publications. After assessing the full text for eligibility, 64 records were excluded. Finally, seventeen studies were included in the pooled analysis (Figure 1).

### 3.1. Characteristics of the Studies

The main features of the eighteen trials included in this meta-analysis are shown in Table 1. Table 2 shows the treatment details. These studies were published between 2013 and 2022; all are multicentre, nine RCTs are European [12,13,14,15,16,17,18,19,20,21,22,23], three are Chinese [24,25,26,27], two are from the USA [28,29,30], one is Iranian [31], two are Korean [32,33], and another is Australian [34].

The eighteen RCTs included 7695 patients, 3829 of whom received intensified chemotherapy as part of the preoperative radiochemotherapy approach. The analysed population of each study varied greatly, ranging from 49 [34] to 1266 [28]. In thirteen studies [12,13,14,15,16,17,18,19,20,21,22,23,24,25,26,27,28,29,30,31,32,33] oxaliplatin was associated with standard radiochemotherapy and in five RCTs [22,23,27,33] there was an irinotecan combination.

### 3.2. Pathological Response

**Figure 2:**
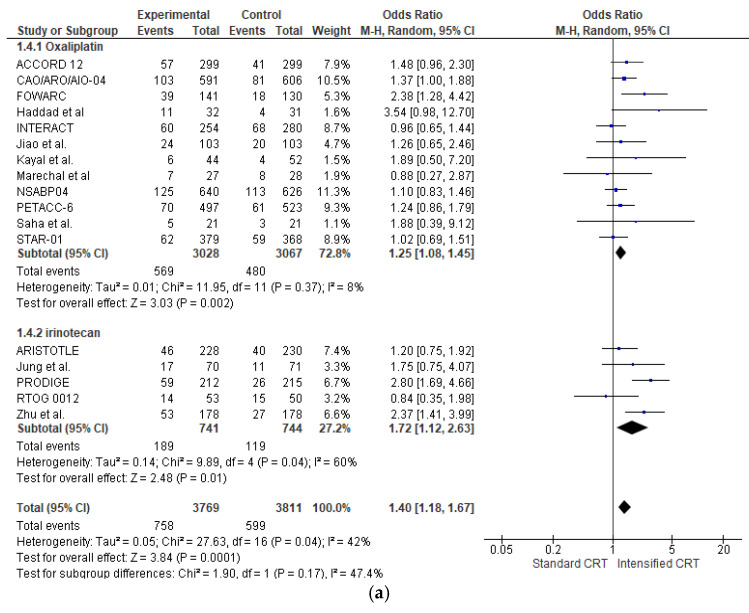

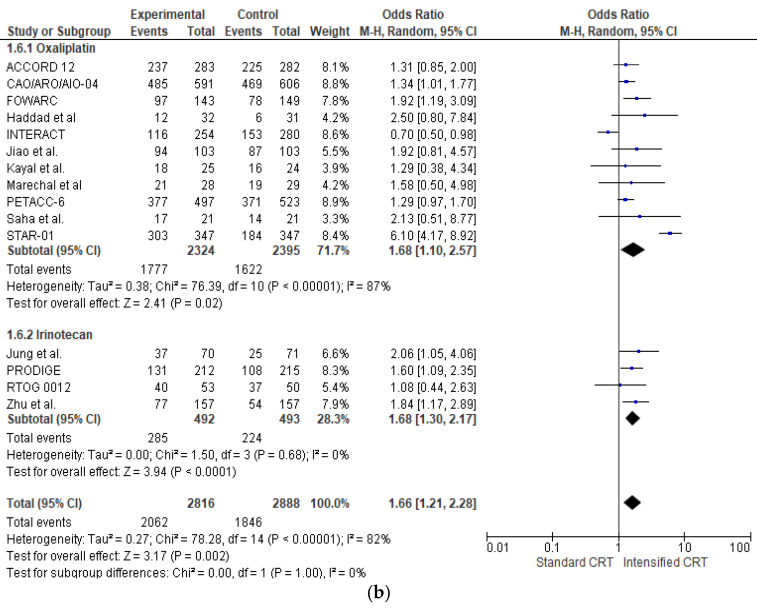
Forest plot of (**a**) pathological complete response and (**b**) pathological major response (sum of complete, near-complete, and moderate regression) included in the meta-analysis obtained using a random effects model. In the random model, the sources of error are both within-study and between-study variance.

The effect of the different preoperative approaches on the complete pathological response (pCR) rate was reported in all RCTs (7580 patients) and is shown in Figure 2a. Adding an intensified chemotherapy with standard radiochemotherapy enhanced pCR in all RCTs but four [17,21,30,34]. However, a statistically significant difference was found in only three studies [23,24,25,27]. The pooled estimate of the treatment effect on pCR was significant (OR 1.40 (95% CI, 1.18–1.67) *p* = 0.0001). There was low heterogeneity between studies for pCR, with an I^2^ = 42%. Globally, 758 patients (20.1%) showed a pCR in intensified chemotherapy CRC versus 599 (15.7%) in the control group.

In the subgroup of RCTs adding oxaliplatin (6095 patients), there was a statistically significant increase in pCR (OR 1.25 (95% CI, 1.08–1.45) *p* = 0.002) with a very low heterogeneity I^2^ = 8%. With “robust analyses”, excluding one study at a time and evaluating 11 RCTs, no significant differences in treatment effect estimates were reported.

In the subgroup of RCTs adding irinotecan (1485 patients), there was a statistically significant increase in pCR (OR 1.72 (95% CI, 1.12–2.83) *p* = 0.04) with a moderate heterogeneity I^2^ = 60%. With “robust analysis”, a lost statistical significance for pCR omitting the Chinese trial by Zhang [27] or Prodige [23], respectively, was demonstrated.

Analysing the effect of two fluoropyrimidines with different administration in capecitabine trials (3704 patients and 675 pCR), there was a statistically significant increase in pCR in the association (OR 1.54 (95% CI, 1.18–2.02) *p* = 0.002) with a low heterogeneity I^2^ = 54%. A similar result is obtained in 5-fluorouracil trials (2610 patients and 444 pCR) (OR 1.33 (95% CI, 1.03–1.71) *p* = 0.03) with a low heterogeneity I^2^ = 19%. There was no significant association between pCR and fluoropyrimidine administration, χ^2^ = 0.96, *p* = 0.62.

The effect of the different preoperative approaches on tumour regression (defined as the sum of patients with complete, near-complete, and moderate regression [35]) was reported in sixteen RCTs (5704 patients) and is shown in Figure 2b. Adding an intensified chemotherapy with standard radiochemotherapy enhanced tumour regression in all RCTs but three [15,16,21,34]. A statistically significant difference was found in six RCTs [14,19,20,23,24,25,27,33]. The pooled estimate of the treatment effect on tumour regression was significant (OR 1.66 (95% CI, 1.21–2.28) *p* < 0.00001). There was high heterogeneity between RCTs with an I^2^ = 82%. In the subgroup of RCTs adding oxaliplatin (4719 patients), there was a statistically significant increase in tumour regression (OR 1.68 (95% CI, 1.10–2.57) *p* < 0.00001) with a high heterogeneity I^2^ = 87%. With “robust analyses”, no significant differences in treatment effect estimates were reported. In the subgroup of RCTs adding irinotecan (982 patients), there was a statistically significant increase in tumour regression OR 1.68 (95% CI, 1.30–2.17) *p* = 0.0001).

Analysing the effect of two fluoropyrimidines with different administration in capecitabine trials (2965 patients and 1898 regressions), there was not a statistically significant increase in pathological regression OR 1.34 (95% CI, 0.99–1.81) *p* = 0.06) with a moderate heterogeneity I^2^ = 66%. In 5-fluorouracil trials (2045 patients and 1523 regressions) (OR 1.51 (95% CI, 1.22–1.86) *p* = 0.0001), with no heterogeneity, there was no significant association between regression and the fluoropyrimidine administration modality, χ^2^ = 1.17, *p* = 0.56.

### 3.3. Disease-Free Survival

**Figure 3:**
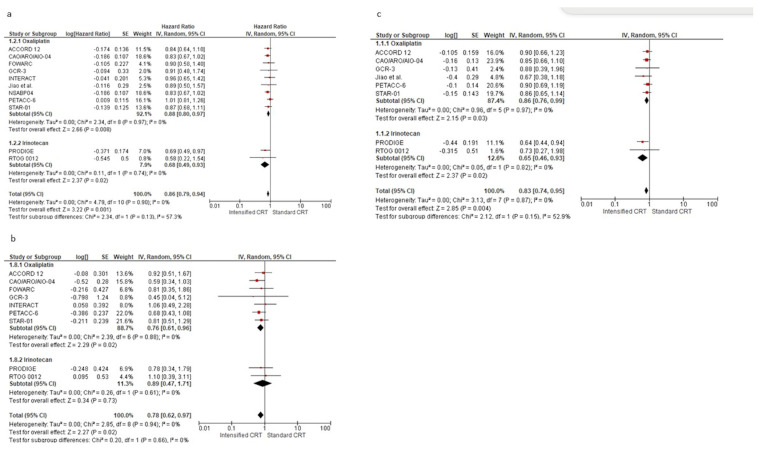
Forest plot of hazard ratios of (**a**) disease-free survival, (**b**) local recurrence-free survival, and (**c**) distant metastases-free survival of RCTs included in the meta-analysis obtained using a random effects model. In the random model, the sources of error are both within-study and between-study variance.

Figure 3a shows the HR for DFS (6618 patients) in twelve RCTs and the overall analysis. The HRs for DFS of an intensified chemotherapy were compared to the control arms in all trials. The effect of treatment on DFS significantly favoured intensified chemotherapy added to standard radiochemotherapy in all trials but two [18,33]. However, a statistically significant difference was observed in no study. Our meta-analysis shows a statistically significant benefit obtained with intensified CRT: the pooled estimate of the treatment effect was significant, HR 0.86 (95% CI, 0.79 to 0.94) *p* = 0.001, corresponding to a 14% reduction of the hazard of disease progression for intensified CRT. No significant heterogeneity was observed between the studies (Χ^2^ = 2.37), I^2^ = 0%. The pooled estimate of the treatment effect was significant when using robust analysis. After omitting the largest trial, NSABP, the evaluation of the remaining ten studies did not lose statistical significance, HR 0.87 (95% CI 0.78–0.96) *p* = 0.006. In RCTs adding oxaliplatin to CRT, there was an 12% reduction of the hazard of disease progression corresponding to a pooled HR of 0.89 (95% CI, 0.80 to 0.97) *p* = 0.008. Instead, pooling RCTs with irinotecan, the HR was 0.68 (95% CI, 0.49 to 0.93) *p* = 0.002. There was no significant association between disease-free survival and the fluoropyrimidine administration modality, X^2^ = 0.28, *p* = 0.87.

After analysing postoperative chemotherapy, no statistical difference was found between studies using 5-FU, with HR 0.89 (95% CI, 0.73 to 1.10) *p* = 0.29. Instead, we found a DFS advantage in studies without a specific recommendation in adjuvant approach, with an HR 0.83 (95% CI, 0.70 to 0.97) *p* = 0.02, and in studies using FOLFOX or CAPOX, with an HR 0.87 (95% CI, 0.77 to 0.99) *p* = 0.04.

The effect of the different preoperative approaches on local recurrence-free survival was reported in nine RCTs (5005 patients) and is shown in Figure 3b. The pooled estimate of the treatment effect on LRFS was significant, HR 0.78 (95% CI, 0.63 to 0.97) *p* = 0.003, corresponding to a 22% reduction of the hazard of local recurrence for intensified CRT. Using oxaliplatin increases local control by about 23%, while irinotecan gives an advantage of 11%. No difference was found according to the two fluoropyrimidines with different administration: capecitabine HR 0.81 (95% CI, 0.59 to 1.10) *p* = 0.17, and 5-fluorouracil HR 0.75 (95% CI, 0.55 to 1.03) *p* = 0.07.

Seven RCTs (4406 patients) evaluated the control of distant metastases, as shown in Figure 3c. The pooled estimate of the treatment effect on DMFS was significant, HR 0.83 (95% CI, 0.73 to 0.95) *p* = 0.005, corresponding to a 17% reduction of the hazard of distant metastases for intensified CRT. No heterogeneity was demonstrated I^2^ = 0%. With “robust analyses”, excluding one study at a time, and therefore evaluating six RCTs, no significant differences in treatment effect estimates were reported.

In the subgroup of RCTs adding oxaliplatin (3876 patients), there was a statistically significant increase in DM control (HR 0.86 (95% CI, 0.75–0.99) *p* = 0.03). In the subgroup of RCTs adding irinotecan (530 patients), there was a statistically significant increase in DM control (HR 0.65 (95% CI, 0.46–0.93) *p* = 0.02). According to the two fluoropyrimidines with different administration, and according to the fluoropyrimidine administration, there was an increased distant metastasis control with capecitabine and oxaliplatin/irinotecan, HR 0.82 (95% CI, 0.69 to 0.97), *p* = 0.02. No difference was seen with 5-fluorouracil, HR 0.85 (95% CI, 0.71 to 1.02) *p* = 0.54.

### 3.4. Survival

**Figure 4:**
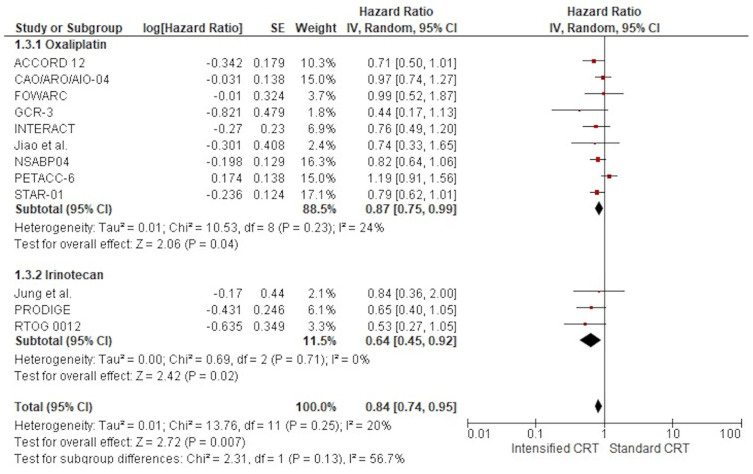
Forest plots of the hazard ratio of RCTs’ overall survival were included in the meta-analysis and obtained using a random effects model.

The effect of adding two chemotherapy drugs to long-term radiotherapy on overall survival (11 studies: 6618 patients) is shown in Figure 4. The treatment effect on OS favoured the two drugs in all but one RCT16; a statistically significant difference was observed in no RCT. The pooled estimate of the treatment effect was significant, with HR 0.84 (95% CI, 0.74 to 0.95) *p*= 0.007, corresponding to a 16% reduction in the risk of death with intensified therapy. A low heterogeneity was observed between the studies (Χ^2^ = 13.76), I^2^ = 20%. The pooled estimate of the treatment effect was significant when robust analysis was used. After removing the largest trial, NSABP, robust analyses showed that all ten remaining studies did not lose statistical significance: HR 0.85 (95% CI 0.73–0.98) *p* = 0.03. In RCTs adding oxaliplatin to CRT, there was a 13% reduction of the risk of death corresponding to a pooled HR 0.87 (95% CI, 0.75 to 0.99) *p* = 0.04. Instead, pooling RCTs with irinotecan, the HR was 0.64 (95% CI, 0.45 to 0.92) *p* = 0.007 (36% reduction of the hazard of disease progression). No difference was found according to the two fluoropyrimidines with different administration: capecitabine had an HR 0.83 (95% CI, 0.68 to 1.00) *p* = 0.05 and 5-fluorouracil HR 0.90 (95% CI, 0.72 to 1.13) *p* = 0.37. There was no significant association between overall survival and the fluoropyrimidine administration modality, χ^2^ = 2.12, *p* = 0.35. Regarding postoperative chemotherapy, no difference was found between studies without a specific recommendation, with HR 0.76 (95% CI, 0.62 to 0.92) *p* = 0.005, and studies using 5-FU, with HR 0.79 (95% CI, 0.64 to 0.97) *p* = 0.02. Instead, we found no OS advantage in studies using FOLFOX or CAPOX as a postoperative treatment, HR 0.97 (95% CI, 0.78 to 1.19) *p* = 0.74.

### 3.5. Toxicity

**Figure 5:**
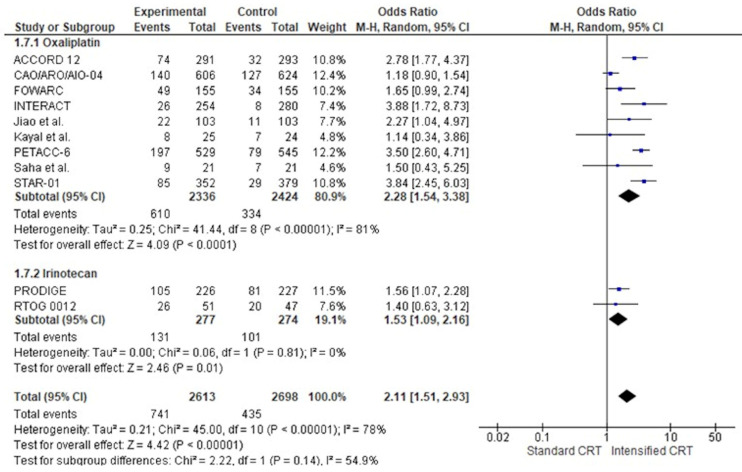
Forest plots of toxicities of RCTs were included in the meta-analysis and obtained using a random effects model.

A severe adverse event (≥G3) was described in ten out of eleven studies, corresponding to 53,365,311 patients, 26,252,613 in the intensified treatment and 27,112,698 in the standard treatment. In all RCTs but one [33], there was an increase in severe adverse events in the double-drug chemotherapy group. The pooled estimate of the adverse events (≥G3) was OR 1.962.11 (95% CI 1.3551–2.8593), *p* < 0.00050001 (Figure 5). Moderate heterogeneity was observed between the studies, with I^2^ = 84%. 78%.

Between severe adverse events (≥G3), the most common were gastrointestinal (8% in the standard CRT and 14% in the CRT with more drugs), haematological adverse effects (4.5% vs. 6%), and genitourinary (0.9% vs. 1.4%). Only three trials [12,13,14], 28 analysed the rate of acute and late toxicities (≥G3). Globally, 17.8% of acute and 11.8% of late (≥G3) adverse effects occurred.

In the subgroup of RCTs adding oxaliplatin, the pooled estimate of adverse events was OR 2.4728 (95% CI 1.35–2.8554–3.38), *p* << 0.00001. In this group, patients treated with intensified chemotherapy with oxaliplatin were two times more likely to have an adverse event ≥ G3.

In the subgroup of RCTs adding irinotecan, the pooled estimate of adverse events was not significantly increased, OR 1.0553 (95% CI 0.501.09–2.19), [16], *p* = 0.89, with no heterogeneity. Analysing the two fluoropyrimidines with different administration, in capecitabine trials (1777 patients and 359 adverse events ≥ G3), there was a statistically significant increase in adverse events ≥G3 in the association with another drug, OR 2.52 (95% CI, 1.77–3.59) *p* < 0.0001 with a moderate heterogeneity, I^2^ = 50%. Instead, a marginal statistical difference was obtained in 5-fluorouracil with an OR 1.71 (95% CI, 1–2.93) *p* = 0.05 with a high heterogeneity of I^2^ = 80%. There was a significant association between adverse events ≥ G3 and the fluoropyrimidine administration modality, χ^2^ = 5.94, *p* < 0.0001.

### 3.6. Publication Bias

**Figure 6:**
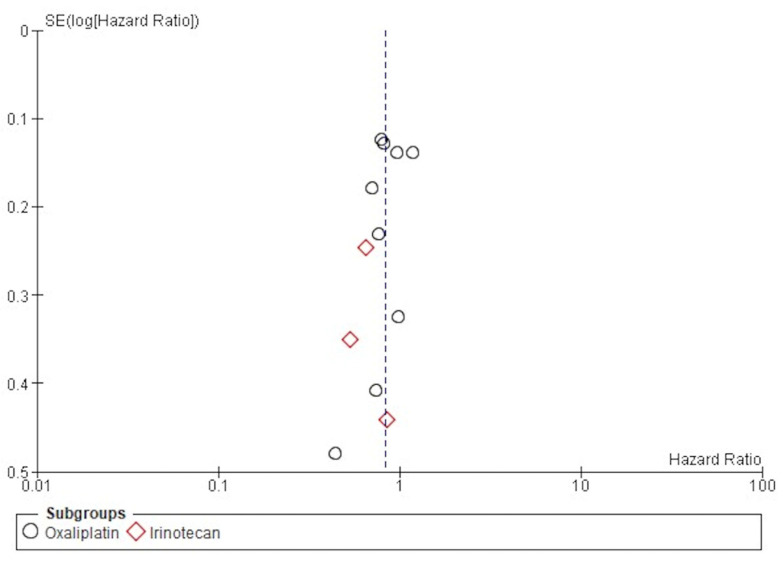
Funnel plot of publication bias for DFS.

The funnel publication bias plot for the OS (Figure 6) and Egger’s test for publication bias showed that the risk of having missed or overlooked trials was not significant (*p* = 0.354).

## 4. Discussion

The results of this meta-analysis demonstrate that adding another chemotherapeutic drug to fluoropyrimidine and long-term radiotherapy can significantly influence all outcomes. Globally, the intensified regimens demonstrated a 16% improvement in OS, a 14% improvement in DFS, a 22% increase in local recurrence control, and a 17% improvement in the control of distant metastases, albeit within a framework of significant heterogeneity among the trials analysed.

At least ten previously published meta-analyses address the benefit of the intensifying chemoradiotherapy approach with another CT added to fluoropyrimidine and RT. Referring to the three most recent [34,35,36], the aggregated data agree, with no difference in overall survival and a statistical increase in distant recurrence control and toxicities. Furthermore, in the most recent 36, it was found to be a significant benefit in terms of DFS. Differing from others, our study also included studies intensifying CRT with irinotecan. Three RCTs had mature data on outcomes: the RTOG 0012 trial, the Prodige trial, and the trial by Jung et al. One by one, these RCTs did not find a statistically significant benefit in OS. Analysing all irinotecan-based studies together, the results reached statistical significance, showing an impressive increase of 36% in OS, 32% in DFS, 11% in local control, and 35% in the reduction of metastatic dissemination.

Furthermore, in this meta-analysis, we have added further studies intensifying with oxaliplatin: the Italian trial INTERACT19. This study considers two approaches: an increase in RT dose (about 10% dose more than standard) and oxaliplatin added to CRT in patients cT2-low-lying/T3 N0-2. The rate of T2 N0 patients is very low, at least 1%, so we can consider this a trial accruing local advanced rectal cancer. Adding this new study and pooling all studies using oxaliplatin, we obtained a statistically significant improvement in OS with an HR 0.87 (95% CI, 0.75–0.99) *p* = 0.04 with a low grade of heterogeneity between studies, I^2^ =24%. All oxaliplatin RCTs, excluding one, show an increase in OS despite no statistical significance. The design of every trial was probably not powered to capture small but meaningful differences in true outcomes. The pooled results also reached statistical significance in DFS with an increase over CRT alone of 12%, local control of 24%, and distant metastases control of 14%.

All analysed RCTs compare the intensification of chemotherapy using a long-term radiotherapy approach. The total radiation dose is homogenous, ranging from 45 to 55 Gy, with a daily dose ranging from 1.8 to 2.2 Gy/day. There were differences in fluoropyrimidine administration: some trials used capecitabine orally and some used 5-fluorouracil in bolus or continuous infusion. We wondered if there were differences in the association between different forms of fluoropyrimidine administration and oxaliplatin and/or irinotecan. All outcomes have no statistically significant difference, excluding a marginally statistically significant increase in adverse events ≥ G3 with 5-fluorouracil. We are not made of the thing considering the results of the NSABP trial [26,27] investigating the impact of adding oxaliplatin to either capecitabine or 5-FU continuous infusion in the neoadjuvant setting. The addition of oxaliplatin did not statistically change the outcome of the trials using oral fluoropyrimidine or continuous infusion.

Similar to other meta-analyses, we found a statistical increase in ≥G3 toxicities, with a global incidence of 25.9% versus 17.6% for standard treatment.

Many efforts have been made to identify the optimal chemotherapy combination that would increase the cost-effectiveness of therapy. There was a considerable variation in the used chemotherapy schedule, suggesting that a standard oxaliplatin or irinotecan schedule is needed to obtain comparable data on the efficacy and safety profile, particularly regarding the dose and the time of CT infusion. Furthermore, the risk of toxic effects is reduced by identifying patients with a genetic profile with a significant risk of adverse events. As with irinotecan dose-associated toxicities related to a UGT1A1×28 genotype [25], it would be necessary to identify a genotype in which the toxic events are increased with oxaliplatin. However, these toxicities are not a significant obstacle to using an intensified CT in combination with radiotherapy. Toxicities are clinically manageable but require careful monitoring. Globally, relevant and speculative information can be obtained by separately evaluating the local control and distant metastases control. We have demonstrated that the probability of achieving improved systemic control with irinotecan-based intensification was notably higher than with oxaliplatin-based intensification and the contrary for local control. It is known that the LARC presents a spectrum of clinical behaviours and biological profiles [37,38]. Some may evolve with a local progression, while others may principally metastasize. A broad arsenal of treatment approaches can ensure that the intervention will be customized to the specific clinical context. With the two intensification regimens, a different modality of action can be used to tailor treatment strategies to patients’ clinical and pathological profiles. Subgroup analysis reveals differing advantages between oxaliplatin and irinotecan regimens: oxaliplatin-based intensification significantly enhances local control (24% improvement), and it could be particularly suitable for patients at high risk of loco-regional recurrence. Factors such as T4b stage, circumferential resection margin (CRM) involvement on MRI, positive lateral pelvic lymph nodes, or tumours within 5 cm of the anal verge are indicative of a disease characterized by local progression [39]. Instead, irinotecan-based intensification, which has a probable higher systemic control (35% improvement in distant metastasis-free survival), can be a better option for patients with a higher likelihood of metastatic progression. An extramural venous invasion EMVI positivity, N2 stage, high tumour budding, and elevated CEA levels are predictors of a higher propensity to metastatic progression of disease. However, a lack of robust clinical or histological markers to stratify patients limits the ability to make definitive treatment recommendations. Emerging biomarkers like cancer/testis antigens (CTAs) NY-ESO-1 and MAGE-A4 have shown significant potential in identifying aggressive tumour behaviour and guiding therapeutic strategies [40]. In a recent study, CTAs were found to be highly expressed in aggressive soft tissue sarcomas, correlating with poor prognosis and aggressive disease phenotypes [41]. These findings suggest that NY-ESO-1 and MAGE-A4 could serve as predictive markers in soft tissue sarcomas and other malignancies with a high metastatic propensity, such as locally advanced rectal cancer [42].

In addition to the highly favourable response observed in LARC with mismatch repair-deficient tumours, which have shown high sensitivity to single-agent PD-1 blockade [43], modifying the tumour microenvironment could offer a promising avenue to improve outcomes in other subgroups. It is becoming clear that beyond the direct cytotoxic effects, chemotherapy agents can also modulate the tumour microenvironment and immune response [44], which may synergize with radiotherapy. Oxaliplatin promotes immunogenic cell death [45], increases the CTL–Treg cell ratio, and depletes myeloid-derived suppressor cells (MDSCs) [46]. Apparent discrepancies were encountered during irinotecan treatments. An MDSC accumulation was shown in the tumour microenvironment [47]. However, experimental evidence highlighted significantly decreased MDSCs at day six after treatment with irinotecan combined with fluorouracil, leading to enhanced tumour-specific responses [48]. These two chemotherapy drugs can modify the density, composition, localization, and function of tumour-infiltrating lymphoid and myeloid cells, modifying the immune context [49] and improving the results obtained by radiotherapy. Immunosuppression cells have an important role in metastatic growth, limiting the ability of MDSCs to migrate into tumours and suppress the immune response, which can increase local radiation treatment and control metastasizing [50].

Radiotherapy, long considered a local treatment, could also have a systemic effect mediated by immune modulation, further supporting the rationale for combination strategies. These findings suggest treatment efficacy depends on tumour cell lethality and interactions with the immune context and microenvironment, as demonstrated in cancer in other sites [51].

The results of this study are subject to several limitations. Differences in the baseline severity of illness in the population of the RCTs, the dose, the type and the combination of CT may limit the accuracy of this meta-analysis. We analysed these differences by including covariates that described the patients studied and the study design features. Finally, we should be particularly concerned about publication bias in settings where relatively small studies are conducted. However, the risk of having missed or overlooked trials in the setting of studies was insignificant when assessed by tests for publication bias. Therefore, small studies with a small treatment effect are unlikely to remain unpublished.

In patients with resectable rectal carcinoma, the available evidence from the literature data is sufficient to conclude that the addition of more chemotherapy drugs to radiotherapy improves OS, but toxicity is significantly increased by adding other chemotherapy drugs to CRT.

## Figures and Tables

**Table 1:** The main features of the trials included in the meta-analysis.

	Simple Size	Mean Age	Male (%)	ECOG 0–1 (%)	cT3 (%)	cT4 (%)	cN+ (%)	Sphincter- Saving Surgery (%)	Definitive Stoma (%)	pCR (%)	ypT0 (%)	ypN0 (%)
**Platin-derived/based**												
ACCORD12 [12,13]	598	62	57	94	87	5	71	69	22	28	17	70
CAO/ARO/AIO-04 [14]	1197	62	71	82	87	7	72	67	25	16	16	69
FOWARC [22,23]	271	53	64	-	62	34	80	-	-	21	-	74
Haddad et al. [31]	63	57	68	-	86	6	89	-	-	24	-	-
INTERACT [19]	534	59	68	-	90	0	77	71	15	29	-	-
Jiao et al. [24]	206	63	62	95	62	36	78	82	18	21	21	67
Kayal et al. [29]	49	-	73	87	31	69	-	71	29	31	-	-
Marechal et al. [15]	57	62	65	100	89	9	90	82	14	26	26	51
NSABP04 [26,27]	1608	-	68	-	-	-	41	74	26	19	-	-
PETACC-6 [16]	1094	62	71	99	85	8	71	67	25	12	-	-
Saha et al. [30]	49	-	73	96	31	69	-	-	28	18	-	-
STAR-01 [17,18]	747	62	67	99	81	<1	65	76	18	-	17	68
**Irinotecan-based**												
ARISTOTLE [20]	564	61	66	-	77	16	-	42	55	20	-	-
Jung et al. [32]	141	57	68	100	79	21	89	95	4	20	20	68
PRODIGE [21]	461	61	66	98	78	16	88	79	13	18	18	69
RTOG 0012 [28]	103	57	65	100	72	30	38	-	-	15	28	-
Zhu et al. [25]	356	55	82	100	80	17	94	56	30	24	25	64

**Table 2:** Chemotherapy and radiotherapy regimens and their dosage of the trials included in the meta-analysis.

	Details of Neoadjuvant Radiation	Details of Neoadjuvant CT		Compliance (%)	≥G3 (%)	Details of Adjuvant CT
**Platin-derived/based**						
		**experimental**	**control**			
ACCORD12 [12,13]	1.8 Gy/25 daily fractions 45 Gy Total	CAPE 800 mg/m^2^ bid × 5 d + OXA 50 mg/m^2^ once per wk	CAPE 800 mg/m^2^ bid × 5 days	94	19	No specific recommendation
CAO/ARO/AIO-0414 [14]	1.8 Gy/28 daily fractions 50.4 Gy Total	5-FU 250 mg/m^2^ per day CVI × d 1–14 and d 22–35 + OXA 50 mg/m^2^ × d 1, 8, 22, and 29	5-FU 1000 mg/m^2^ per day CVI over 5 d × d 1–5 and d 29–33	75	23	5-FU/LV/OX: OXA 100 mg/m^2^ × d 1 and 15 + LV 400 mg/m^2^ × d 1 and 15 + 5-FU 2.4 g/m^2^ over 46 h CVI d 1–2 and d 15–16
FOWARC [22,23]	1.8 Gy/28 daily fractions 50.4 Gy Total	5-FU 225 mg/m^2^ per d × 5 days CVI + OXA 60 mg/m^2^ per wk	5-FU 225 mg/m^2^ per d × 5 days CVI	90	27	Fluorouracil-based adjuvant chemotherapy
Haddad et al. [31]	1.8 Gy/25 daily fractions + boost of 5.4 Gy/3 fractions 50.4 Gy Total	CAPE 825 mg/m^2^ bid × 5 d + OXA 60 mg/m^2^ once per wk × 5–6 cycles	CAPE 825 mg/m^2^ bid × 5 days	100	13	No specific recommendation
INTERACT [19]	1.8 Gy/25 daily fractions + boost of 5.4 Gy/3 fractions 50.4 Gy Total	CAPE 1300 mg/m^2^ in three time/day × 7 days + OXA 130 mg/m^2^ d 1, 19, and 38	CAPE 1650 mg/m^2^ in three time/day × 7 days	96	6	5-FU-based
Jiao et al. [24]	2 Gy/25 daily fractions 50 Gy Total	CAPE 800 mg/m^2^ bid × d 1– 14 and d 22– 25 + OXA 60 mg/m^2^ d 1, 8, 22, and 29	CAPE 800 mg/m^2^ bid. × d 1–14 and d 22–25	83	16	mFOLFOX6: OXA 85 mg/m^2^ + LV 400 mg/m^2^ + 5-FU 400 mg/m^2^ bolus+ 5-FU 2.4 g/m^2^ over 46–48 h CVI every 2 wk × 6–8 cycles
Kayal et al. [29]	1.8 Gy/28 daily fractions 50.4 Gy Total	5-FU 350 mg/m^2^ CVI × d 1–5 and d 29–33 + CIS 100 mg/m^2^ × d 1 and 29	5-FU 350 mg/m^2^ CVI × d 1–5 and d 29 + LV 20 mg/m^2^	-	29	mFOLFOX6: OXA 85 mg/m^2^ + LV 400 mg/m^2^ + 5-FU 400 mg/m^2^ bolus + 5-FU 2.4 g/m^2^ over 46–48 h CVI every 2 wk× 4
Marechal et al. [15]	1.8 Gy/25 daily fractions 45 Gy Total	day 1 and 14: 5-FU 400 mg/m^2^ on day 1 with folinic acid 400 mg/m^2^ on day 1, 5-FU 2000 mg/m^2^ CVI over a 46 h period CVI + OXA 100 mg/m^2^ i.v. over 2 h	5-FU 225 mg/m^2^ per d × 5 days CVI	97	21	No specific recommendation
NSABP04 [26,27]	1.8 Gy/25 daily fractions/5 wk + boost of 5.4 Gy/3 fractions (boost of 10.8 Gy/3 fractions for T4 or fixed distal tumours) 50.4 Gy (55.8 Gy) Total	5-FU 225 mg/m^2^ per d × 5 days CVI + OXA 50 mg/m^2^ once per week or CAPE 825 mg/m^2^ bid × 5 days + OXA 50 mg/m^2^ once per week	5-FU 225 mg/m^2^ per d × 5 days CVI or CAPE 825 mg/m^2^ bid × 5 days	-	33	No specific recommendation
PETACC-6 [16]	1.8 Gy/25 daily fractions + optional boost of 5.4 Gy/3 fractions (d 36–38) 45 Gy/50.4 Gy	CAPE 825 mg/m^2^ bid × d 1–33 w/o weekends + OX 50 mg/m^2^ × d 1, 8, 15, 22, and 29	CAPE (825 mg/m^2^ bid × d 1–33 w/o weekends)	92	48	CAPOX: CAPE 1000 mg/m^2^ bid × d 1–15 + OXA 130 mg/m^2^ × d 1 every 3 wk for 6 cycles
Saha et al. [30]	1.8 Gy/28 daily fractions 50.4 Gy Total	CAPE 1000 mg/m^2^ bid × d 1–14 and d 25–38 + OXA 85 mg/m^2^ × d 1 and 29	5-FU 350 mg/m^2^ CVI × d 1–5 and d 29 + LV 20 mg/m^2^	-	43	mFOLFOX6: OXA 85 mg/m^2^ + LV 400 mg/m^2^ + 5-FU 400 mg/m^2^ bolus + 5-FU 2.4 g/m^2^ over 46–48 h CVI every 2 wk × 4
STAR-01 [17,18]	1.8 Gy/28 daily fractions 50.4 Gy Total	5-FU 225 mg/m^2^ per d × 5 days CVI + OXA 60 mg/m^2^ per wk	5-FU 225 mg/m^2^ per d × 5 days CVI	83	15	Fluorouracil-based adjuvant chemotherapy
**Irinotecan-based**						
ARISTOTLE [20]	1.8 Gy/25 daily fractions 45 Gy Total	CAPE 650 mg/m^2^ bid + weekly Irinotecan 60 mg/m^2^ weeks 1–4	CAPE 900 mg/m^2^ bid × 5 days	72	16	No specific recommendation
Jung et al. [32]	1.8 Gy/28 daily fractions 50.4 Gy Total	Irinotecan 40 mg/m^2^ on days 1, 8, 15, 22, and 29 + S-1 70 mg/m^2^ on the day of irradiation	5-FU (400 mg/m^2^/day)+ LV 20 mg/m^2^/day for 3 consecutive days every 4 weeks for 2 cycles	86	6	Fluorouracil-based adjuvant chemotherapy
PRODIGE [21]	2 Gy/25 daily fractions 50 Gy Total	OXA 85 mg/m^2^ + LV 400 mg/m^2^ (2 h ev) followed by Irinotecan 180 mg/m^2^ (90 min ev) and 5-FU 2400 mg/m^2^ CVI over 46 h. FOLFIRINOX was given every 14 days for six cycles.	CAPE 800 mg/m^2^ bid × 5 days	89	60	mFOLFOX6: OXA 85 mg/m^2^ + LV 400 mg/m^2^ + 5-FU 400 mg/m^2^ bolus + 5-FU 2.4 g/m^2^ over 46–48 h CVI every 2 wk × 4
RTOG 0012 [28]	1.8 Gy/25 daily fractions (+boost 5.4 T3 and 10.8 T4) 50.4–60.4 Gy Total	5-FU 225 mg/m^2^ per d × 5 days CVI and weekly 50 mg/m^2^ Irinotecan	5-FU (225 mg/m^2^ per d × 5 days CVI)	100	13	no specific recommendation
Zhu et al. [25]	2 Gy/25 daily fractions 50 Gy Total	CAPE 625 mg/m^2^ bid 5 d/wk and weekly 80 mg/m^2^ Irinotecan, followed by a cycle of CAPE 1000 mg/m^2^ bid plus Irinotecan 200 mg/m^2^ (XELIRI) 2 weeks after CRT	CAPE (825 mg/m^2^ bid × 5 days)	99	22	CAPOX (CAPE 1000 mg/m^2^ bid × d 1–15 + OXA 130 mg/m^2^ × d 1 every 3 wk for 6 cycles)

## Supplementary Materials

The following supporting information can be downloaded at: https://www.mdpi.com/article/10.3390/jcm14020345/s1, Figure S1: Quality assessment of included studies.

## Abbreviations

LARC, locally advanced rectal cancer; CRT, chemoradiotherapy; 5-FU, 5 fluorouracil; RCT, randomised controlled trial; pCR, pathological complete response; DFS, disease-free survival; OS, overall survival.
