# Supplementary material for: “Add More Arrows to Your Quiver”: The Role of Adding Another Chemotherapy Drug to Fluoropyrimidine and Long Term Radiotherapy in Locally Advanced Rectal Cancer: A Systematic Review and Meta-Analysis
Source: J Clin Med. 2025 Jan 8;14(2):345. doi: 10.3390/jcm14020345 (PMC11765640; doi:10.3390/jcm14020345)
Supplement: Supplementary file 1 [file jcm-14-00345-s001.zip › jcm-3354916-supplementary.pdf]

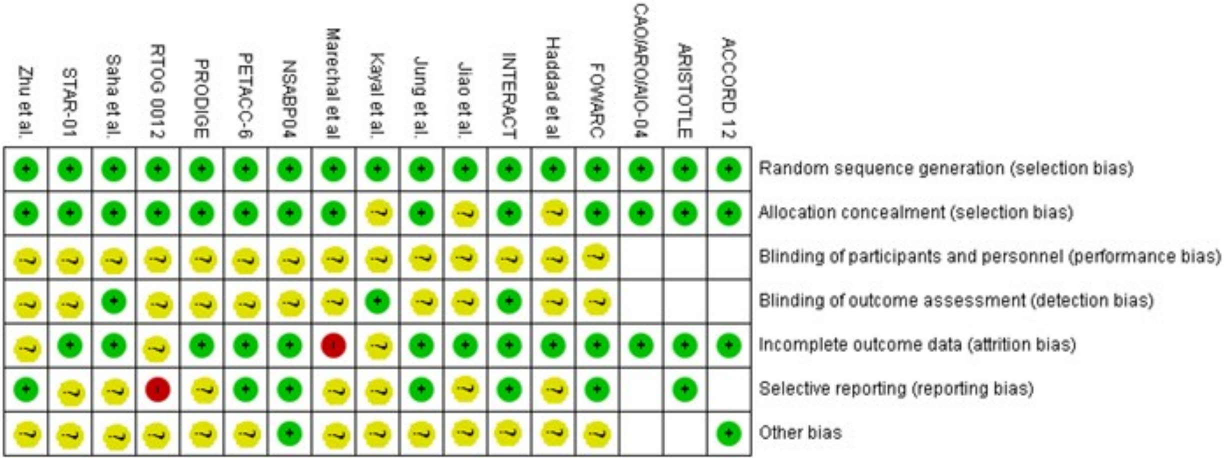
Zhu et al.
STAR-01
Saha et al.
RTOG 0012
PRODIGE
PETACC-6
NSABP04
Marechal et al
Kayal et al.
Jung et al.
Jiao et al.
INTERACT
Haddad et al
FOWARC
CAO/ARO/AIO-04
ARISTOTLE
ACCORD 12
Random sequence generation (selection bias)
Allocation concealment (selection bias)
Blinding of participants and personnel (performance bias)
Blinding of outcome assessment (detection bias)
Incomplete outcome data (attrition bias)
Selective reporting (reporting bias)
Other bias
